# Modeling Emotional Valence Integration From Voice and Touch

**DOI:** 10.3389/fpsyg.2018.01966

**Published:** 2018-10-12

**Authors:** Yacine Tsalamlal, Michel-Ange Amorim, Jean-Claude Martin, Mehdi Ammi

**Affiliations:** ^1^LIMSI, CNRS, Univ. Paris-Sud, Université Paris-Saclay, Orsay, France; ^2^CIAMS, Univ. Paris-Sud, Université Paris-Saclay, Orsay, France; ^3^CIAMS, Université d’Orléans, Orléans, France

**Keywords:** voice expressions, tactile stimulation, emotional valence, multisensory information integration, affective computing

## Abstract

In the context of designing multimodal social interactions for Human–Computer Interaction and for Computer–Mediated Communication, we conducted an experimental study to investigate how participants combine voice expressions with tactile stimulation to evaluate emotional valence (EV). In this study, audio and tactile stimuli were presented separately, and then presented together. Audio stimuli comprised positive and negative voice expressions, and tactile stimuli consisted of different levels of air jet tactile stimulation performed on the arm of the participants. Participants were asked to evaluate communicated EV on a continuous scale. Information Integration Theory was used to model multimodal valence perception process. Analyses showed that participants generally integrated both sources of information to evaluate EV. The main integration rule was averaging rule. The predominance of a modality over the other modality was specific to each individual.

## Introduction

Introducing emotion into Human–Computer Interaction (HCI) and Computer–Mediated Communication (CMC) is becoming an important opportunity with much potential. In fact, emotions constitute a prominent phenomenon in human life. They influence our perceptions, the way to communicate and how we make decisions (Lewis et al., 2010). Researchers in Affective Computing propose to study and design systems that recognize, process, and simulate human affects (Scherer et al., 2010). Emotion communication involves several non-verbal communication channels that can be used separately or simultaneously. Most research in Affective Computing has focused on the study of visual and audio signals. Haptic cues have been neglected even though recent research in psychology has demonstrated that touch is a powerful means of detecting and displaying emotions (Patel, 2013). Recently, haptic platforms were developed in order to maintain physical contact between remote people or between humans and autonomous agents like virtual avatars or robots (Haans and IJsselsteijn, 2005). Haptic devices exploit two types of haptic feedback: tactile and kinaesthetic feedback. The associated research has focused on the qualitative evaluation of these devices, for instance, the study of the usefulness of haptic platforms during interpersonal communications. However, very few studies have investigated how the generated haptic cues are perceived by users in these contexts (HCI and CMC) (Haans and IJsselsteijn, 2005). Modeling the relationship between the physical features of the stimulation and the emotional percept from haptics among participants is crucial for designing credible signals for HCI and CMC platforms. Such models may increase the communication capacities with telepresence systems, and the emotional expressivity of virtual agents, by effectively introducing a sensory channel that can physically convey additional information related to the social and emotional messages. In particular, these models could be used to haptically modulate the perception of emotions conveyed with another sensory channel. For instance, emotional dimensions (e.g., valence) of the facial expression of a virtual agent could possibly be modulated by suitable haptic feedback.

Based on an interdisciplinary approach (psychology, neuroscience, and computer science) inspired by previous research in Affective Computing (Scherer et al., 2010), this paper investigates how tactile and audio sources of information displayed by a system are combined and weighted by participants in the context of an emotional HCI interaction. We focus on modeling the integration mechanisms underlying the combination of voice and touch information in the perception of Emotional Valence (EV). EV is one of the major three dimensions that constitute the theoretical model of emotion called PAD (Russell and Mehrebian, 1977). This model describes emotions using three uncorrelated and continuous bipolar dimensions (i.e., scales): Pleasure (P, also called Valence): degree of well-being (unpleasant – pleasant); Arousal (A): degree of mental or physical activity (relaxed – activated); and Dominance (D): degree of control of a situation (feeling of being controlled – being in control). Modeling voice and touch integration of emotional cues by future users along these dimensions should enhance the design of future multimodal mediated affective platforms.

In the presented experiment, audio stimuli consisted of acted emotional speech extracted from a validated corpus (Bänziger et al., 2012). Tactile stimulation was designed in a previous study and exploits a novel tactile stimulation strategy based on an air jet system (Tsalamlal et al., 2013). This stimulation strategy does not require any physical contact between the participant and the hardware device. The interface can be used to stimulate different and large areas on the body (e.g., the whole forearm). Moreover, it is possible to generate low amplitude forces, which might be more efficient for stimulating mechanoreceptors involved in tactile affective communication. The design of the tactile stimuli consisted of identifying the physical parameters of the air jet stimulation (i.e., physical intensity and speed (Tsalamlal et al., 2015).

During the experiment, participants were asked to evaluate the EV of unimodal (voice or tactile) and bimodal (voice combined with tactile) stimuli. Using the collected data, we studied how participants perceived and integrated bimodal emotional cues within a framework called Information Integration Theory (IIT) (Anderson, 1981).

The rest of the paper is organized as follows. The review section addresses both the psychological and technological aspects of emotion communication with haptic feedback. In Section “Theoretical BASES”, we introduce the theoretical concepts involved in this study, including the PAD model of emotion and IIT. Section “Materials and Methods” describes the protocol of the experiment and Section “Results” discusses the results. The last section highlights future directions and concludes the paper.

## Literature Review

### Display and Perception of Emotions

Communication of emotions through facial expressions has been widely studied (most often for basic emotion categories) (Ekman and Friesen, 1975; Carroll and Russell, 1996; Adolphs, 2002). It was observed that people effectively express and recognize different distinct emotions using this modality. Other studies have shown that some emotions and their dimensions can also be conveyed through other channels. For instance, voice is an effective modality to convey some cues related to arousal, valence and specific emotion categories through acoustic properties (Scherer, 2003; Callaway and Sima’an, 2006). Many studies have investigated acoustic features that describe or categorize emotion vocal expressions. Most acoustic parameters are derived from fundamental frequency (F0), amplitude, duration, or measures derived from spectral analyses (Pierre-Yves, 2003). Touch has received less attention than have facial expression or voice. Recently, researchers have nevertheless showed that touch effectively supports affective cues. For example, Bailenson et al. (2007) conducted two experimental studies to highlight the physical features engaged in the haptic expression of different emotions. In the first experiment, participants were asked to use a joystick with two degrees of freedom/force-feedback to express seven emotions. In the second experiment, a group of participants was asked to recognize expressions of emotions recorded during the first experiment. The results showed variance in handshaking behavior when different emotions were being expressed and this variance can be quantified in meaningful ways. For example, sadness was expressed in slow, steady, and short movements, whereas joy was expressed in long, jerky, and fast movements. Also, people were able to recognize the emotions expressed above the chance level using the device. Using the same type of haptic device but with three degrees of freedom, Gaffary et al. showed that the intensity and duration of the stimuli can discriminate between the expression of anger and joy (Gaffary et al., 2013). Other works explored the hedonic aspects of tactile stimulation. They demonstrated evidence of unmyelinated CT-fibers with a particular potential to elicit pleasant or unpleasant subjective perceptions (McGlone et al., 2007; McCabe et al., 2008; Olausson et al., 2010; Ackerley et al., 2013; Gordon et al., 2013).

In everyday life, people may communicate emotion using different channels simultaneously. For example, we may display facial expression of happiness and talk with prosody expressing joy. Recently, a few studies have explored the multimodal aspect of affective expressions and perceptions. The majority of these studies considered the combination of face and voice (Campanella and Belin, 2007). The results of these studies suggest that congruency in information between facial expression and affective prosody facilitates behavioral reactions to emotional stimuli (Massaro and Egan, 1996; Dolan et al., 2001; Edwards et al., 2002; Shackman and Pollak, 2005). Also, the information obtained via one modality may influence the information processing in another modality (de Gelder and Vroomen, 2000; Ethofer et al., 2006). Generally, these studies observed that facial expression is more important than is voice for judging a portrayed emotion. However, App et al. (2011) investigated the hypothesis that different emotions are most effectively conveyed through specific non-verbal channels of communication. The authors stated that the social function of an emotion predicts its primary channel. The body might promote social status emotions, the face might support survival emotions, and touch might support intimate emotions.

Some studies have considered touch combined with other modalities for the expression and perception of emotions (Bickmore et al., 2010; Gaffary et al., 2014). The authors of these studies found that participants based their judgment on facial expressions to evaluate the valence of emotions, whereas the haptic modality was used to assess the arousal and dominance dimensions of the emotions. Surprisingly, there is no work that addresses the combination of voice and touch in the course of emotion perception. Modeling this combination process is crucial for designing efficient systems that are able to recognize and display affective cues from touch and audio streams. For example, during human–robot interaction, robots could exploit audio and haptic signals to recognize the emotion displayed by the user. To capture these signals, the robot must be equipped with audio and touch (i.e., tactile, force) sensors. Furthermore, in return, the robot may display affective messages using touch and audio feedback simultaneously. In the context of the design of a platform for multimodal communication of emotions, the current paper proposes studying and modeling the integration process of emotion perception of a tactile stimulation and voice emotional expression. Our approach consists in: (1) defining a theoretical model of emotion to measure subjective perception of voice and touch expressions, (2) defining the appropriate framework for modeling the combination process of voice and touch, and (3) conducting an experimental study to highlight how a group of participants combine and weight bimodal cues for the perception of emotion.

### Haptic Devices for Emotion Communication

Several studies have proposed the use of existing haptic interfaces or novel devices for social and emotional communication. This section presents a review of those devices for both long distance interpersonal communication and interaction with autonomous agents.

#### Mediated Interpersonal Communication With Touch

Many studies have investigated the use of haptics for mediated communication with both kinaesthetic and tactile feedback. Tactile perception involves the cutaneous senses. It allows us to feel contact with objects, textures, reliefs or even the rigidity of an object. However, the tactile devices are more popular than kinaesthetic devices, probably because actuation technologies like tactile-vibrators are easier to integrate into portable systems. It was observed that vibration could help users to convey presence, emotion, and empathy during mediated interaction. The devices based on vibrotactile simulation may have many forms. For example, the CheekTouch device (Park, 2010) provides tactile–mediated communication via a mobile phone. The tactile feedback is applied on the user’s cheek and corresponds to the remote partner’s multi-finger input expressed on another mobile phone. The authors designed different affective touch behaviors like pinching and stroking. The user study suggested that this tactile stimulation technique was positively evaluated. The TaSSt (Huisman et al., 2013) is a tactile device that enables two people to communicate different types of touch at a distance. The first part of the device is a touch-sensitive surface where a user can express patterns of touch. The second part is composed of a grid of vibrotactile actuators that are able to render the patterns to the remote partner. An initial evaluation of the device revealed that this approach was capable of displaying some touch patterns like pressing and poking. Using the same type of display but covering a larger surface, a teleconferencing system named The HugMe platform (Cha et al., 2008) enables the haptic feedback to convey affection and intimacy. A suit that embeds vibrotactile actuators provides the haptic stimulation. An active user can see and touch a remote passive user. Some research investigates other tactile stimulation techniques. For instance, the UltraHaptics (Obrist et al., 2015) exploits ultrasound transducers to generate focused air pressure waves on the human hand. This mid-air tactile stimulation technique was used to design an emotional tactile stimulus by asking a group of participants to control the parameters of the tactile stimulation from the device according to a series of displayed pictures. The assessment of the tactile stimulus by a second group shows that this approach communicates well the emotional arousal, but less the EV.

Kinaesthetic-based systems are less popular. These types of devices cannot be portable and may present physical constraints that limit the quality of the interaction. For example, Alhalabi and Horiguchi (Alhalabi Osama and Susumu, 2001) used PHANToMs haptic arms to design the Tele-Handshake platform that enables people to touch and shake hands from a distance. The user study showed that even if the forces were accurately transferred between partners, the handshake was not perceived as realistic. Tsetserukou and Neviarouskaya (2010) proposed a novel system to enhance emotional cues during mediated communication in videogames and virtual environments (ex., Second Life). The system was based on a model of affect analysis that automatically recognizes emotions from text. Then, the identified emotion is communicated through a haptic device by simulating a human hug.

#### Touch Communication With Autonomous Agent

Today, many H–CI applications involve using intelligent agents to provide social presence. These agents can express different aspects of emotions mainly using facial expression, gesture, and speech. Recently, different studies have investigated social and affective touch interactions between humans and virtual agents or social robots. For example, Bickmore et al. (2010) designed a virtual agent capable of physically touching users in synchrony with other non-verbal channels of communication. The agent was composed of an animated human like face displayed on a monitor fixed on the top of a human mannequin. Touch behaviors were conveyed via an air bladder that squeezes a user’s hand. The authors observed that when touch is used in the context of an empathic and comforting interaction, there is a better perception of the relationship with the agent. Mitsunaga et al. (2006) developed a human-size humanoid robot called “Robovie-IV.” The robot has the capability to interact with users via different channels: voice, gesture, and haptic. It was equipped with layers of tactile sensors embedded in a soft skin in order to make it sensitive to haptic interactions. These authors conducted a long-term experiment in their office to evaluate and enhance the interaction abilities of the robot. Based on human–animal interaction studies, Yohanan and MacLean (2011) developed a robot that mimics a small pet interacting with users through touch. Using different tactile sensors, the robot can measure the touch patterns displayed by users and execute some behavior. The authors conducted an experimental study to determine the patterns that participants would likely use when conveying different emotions. The results documented which gestures and physical properties the human was more likely to use and for which specific emotions. The “Probo” is another animal-like robotic companion capable of active relational and affective touch–based interactions (Saldien et al., 2008). This huggable device was developed to increase the wellbeing of hospitalized patients. An experimental study was conducted to highlight the role of this social robot in robot-assisted therapy with autistic children. The results showed that, in specific situations, the social performance of autistic children improves when using the robot Probo as a medium for social storytelling compared to when a human reader tells the stories (Vanderborght et al., 2012). In the field of Human–Robot Interaction, some research specifically focused on the design of devices and control algorithms that generate humanlike handshake interactions (Avraham et al., 2012). Ammi et al. (2015) conducted a study to highlight how the physical features of a robot handshake (i.e., exerted force, stiffness of movement) influence the participants’ perception of a robot’s facial expressions. The results related to the multimodal condition clearly showed that introducing high values for grasping force and stiffness of movement for the three investigated emotions leads to the increase of the perceived arousal and dominance compared to a visual-only condition.

The design of systems that include touch as a means of mediated affect communication is still at its starting age. Researchers have to face methodological constraints related to the study of human touch interactions, in addition to the technical aspects. The majority of actual haptic devices must be physically connected to the user through mechanical systems. These systems are often intrusive, limiting the comfort and the transparency of the interaction, especially in the context of affective communication. Based on the results of some relevant studies highlighting the role of touch in the perception of social and affective behaviors, here we investigate a new form of tactile stimulation for affective communication. This technique does not require any physical contact with any structure. It can be used to stimulate different and large areas on the body (e.g., the whole forearm) in safety. Our air jet system permits generating continuous low amplitude forces that can be especially effective in activating tactile afferents (Gordon et al., 2013). In a previous study (Tsalamlal et al., 2013), we highlighted the fact that manipulating the air jet stimulation parameters enabled the participants to discriminate affective perceptions. More precisely, this work showed a strong link between the intensity of the air jet tactile stimulation and the perception of valence, arousal and dominance dimensions. When the intensity is high, the tactile stimulus is perceived unpleasant, dominant with a high arousal, and when the intensity is low, the tactile stimulus is perceived pleasant with low arousal. Generally, touch is used simultaneously with other modalities to communicate affective messages. In the study described in this paper, we explore how touch is combined with voice over the course of the perception of EV.

## Theoretical Bases

### PAD: A Dimensional Model of Emotion

One goal of our research is to build a computational model of bimodal emotion perception. This model could be implemented in future autonomous agents capable of the recognition and display of combined voice and touch emotional cues. In consequence, it is essential to select a theory of emotion that allows for the implementation of such models. In the literature related to the study of emotions, we find different psychological theories (ex., discrete emotion theories, dimensional theories, and appraisal theories) that are relevant for designing affective computing systems (Scherer et al., 2010). This diversity of approaches to emotion reflects the complexity of emotional phenomena. For our research, the dimensional theories of emotion appear to be most appropriate. These theories argue that emotions can be represented or discriminated by their position in a continuous dimensional space such as the 3D PAD model.

These dimensions corresponds to Pleasure (or valence measure), Arousal (or level of activation measure), and Dominance (or control measure) (Bradley, 1994; Cao et al., 2008). To build our model, we focus on the relationship between the physical parameters of audio streams and tactile stimulations, and the corresponding subjective emotional perception. In dimensional approaches to emotion, emotion is represented on continuous scales (dimensions), and thus can be mapped on continuous physical features, like tactile stimulation (e.g., airflow rate) or an audio stream (e.g., fundamental frequency, F0). Manipulating continuous dimensions (compared to emotion categories) should facilitate the design of the computational mode for audio–haptic communication.

### Information Integration Theory: A Framework for Modeling the Integration of Audio and Touch Stimulation

Norman H. Anderson (Zalinski and Anderson, 1989; Anderson, 1996) proposed the IIT to describe and model how a person evaluates and integrates information from two or more sources to make an overall judgment. The theory focuses on evaluating the unobservable psychological processes involved in making complex judgments.

**FIGURE 1:**
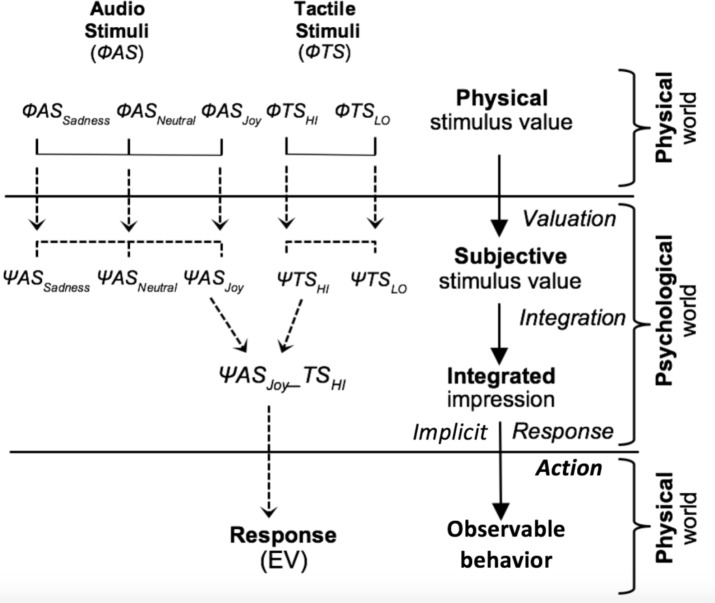
Schematic diagram of the Information Integration Theory, adapted from Anderson (1996).

The IIT was developed around three psychological processes (or functions): valuation, integration, and action (response production). The psychological structure of this integration approach is illustrated in **Figure 1**. The valuation function V corresponds to the transformation of the physical stimulus value (For example, Φ*TS_HI_*) into a subjective value (here Ψ*TS_HI_*) mapped on the response scale (here from -100 to 100). This valuation is operated separately for each informational source. These psychological stimuli are combined by the integration function I (For example, Ψ*TS_HI_* and Ψ*AS_Joy_* combined into Ψ*TS_HI_-AS_Joy_*) to yield an implicit psychological response that is then transformed by the response function R into an observable response measure (here, EV).

Functional measurement was developed as a part of IIT to identify the integration function I (Anderson, 1982, 1971; Zalinski and Anderson, 1989; Oliveira et al., 2005). It uses a class of algebraic rules (cognitive algebra) to model this integration function: adding, multiplying, averaging (with equal or differential weighting), etc. Functional measurement lies in the experimental study of the cognitive algebra. In other words, it is the methodology used to investigate the theoretical framework (an algebraic model of judgment) provided by IIT. Accordingly, based on the graphical pattern displayed by the empirical data and the statistical analyses, one can determine the rules displayed by participants to integrate sources of information to evaluate a complex phenomenon.

A central distinction in IIT is that between value and weight. As Anderson (1971) put it: “Each piece of information is represented by two parameters: a scale value, s, and a weight, w. The value is the location of the informational stimulus along the dimension of judgment. The weight represents the psychological importance of the information. It is important to note that both s and w will depend on the dimension of judgment as well as the individual” (p.172). In model terms, valuation comprises the determinants and the measurement of the w and s parameters, whereas integration involves the ways in which stimuli are combined.

IIT has been used to explain emotion recognition of naturalistic expressions (Oliveira et al., 2007; Courbalay et al., 2016; Pereira et al., 2016; Silva and Oliveira, 2016). Most of the studies used realistic virtual human characters allowing for precise control of facial expression and body posture. It has been shown that, in static facial expressions, the different activated pain relevant muscles are integrated visually using summative-subtractive rules when judging either “expressed intensity,” “naturalness of the pain expression,” “dosage of analgesia required to stop pain,” or “dosage of analgesia required, also accounting for the trustworthiness of the expression” (Oliveira et al., 2007). In contrast, when combining sources of information carried by different body parts, such as facial expression and body posture, averaging seems to be the predominant integration rule (Courbalay et al., 2016; Silva and Oliveira, 2016). It is the case when judging the intensity, valence, or arousal of the combination of face and body expressing basic emotions (e.g., happiness, anger, and sadness) or so-called social self-conscious emotions such as shame and pride (Courbalay et al., 2016). Averaging is also prevailing when estimating back pain intensity from the facial expression and body posture of virtual character performing a trunk flexion–extension movement (Prigent et al., 2014).

In the next sections, we explain how we used this theory for exploring the perception of audio and haptic expressive stimuli.

## Materials and Methods

### Objective

We conducted an experimental study to highlight the integration processes of air jet tactile stimulation together with voice expressions over the course of perceiving EV. We focused on the valence dimension instead of arousal or dominance because valence has not yet been thoroughly researched but nevertheless seems promising and important for H–CI.

The experimental protocol was based on the IIT framework. We presented a set of stimuli to a group of participants and recorded their rating of EV. The collected data were analyzed using functional measurements.

### Participants

23 participants (18 males and five females aged between 21 and 55 years old) took part in this study. All participants were right-handed. None of the subjects had neurological or physical injuries that would affect the sensitivity of the arm or audition. Subjects gave informed consent prior to testing, and the institutional internal review board of the laboratory (IRB) approved this study design.

### Experimental Platform

**FIGURE 2:**
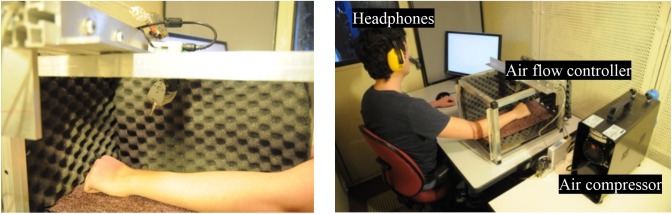
Experimental platform. Participants perceived tactile stimuli on the forearm with the air jet system and audio stimuli (voice expressions) with headphones.

Two types of stimuli were presented to the participants: (1) voice expressions and (2) air jet tactile stimulations. Voice expressions consisted of pseudo speech sentences uttered with prosody. The voices were synthesized and then subjected to a morphing operation to obtain different levels of prosody. Section “Studied Conditions and Stimuli” details the methodology to generate the audio stimuli. The expressions were presented using headphones (SENNHEISER HD 280 PRO). The tactile stimulation was presented using the air jet system (Silva and Oliveira, 2016). This tactile system comprised a rotating air nozzle and airflow regulator. The air nozzle was actuated with a motor controlled in position and velocity. The rotation of the nozzle was controlled at the motor axis. This rotation enabled the diffusion of air along the arm of the participant (**Figure 2**). The flow controller accurately regulated the flow rate of the outlet air jet (MFC Bukert 8711, up to 50l/min ± 0.02). An air compressor provided sufficient air pressure to the system (i.e., four bars). The nozzle, mounted on a motor, was placed inside a box where the participant laid his or her arm on a support. A software triggered the tactile stimuli and the audio samples. After each stimulus, participants rated its EV using a track bar displayed on the screen that was controlled by a mouse (see **Figure 2**).

### Hypotheses

Based on existing studies evaluating EV of tactile and audio stimuli (see section “Literature Review”), we formulated several hypotheses.

**H1. Unimodal estimations:**

(A) The levels of tactile stimulation are well-discriminated on the EV dimension.
(B) The levels of audio stimulation are well-discriminated on the EV dimension.

**H2. Bimodal estimations:**

(A) Participants base their judgments on both modalities (weighting each information) to evaluate EV.
(B) Participants give higher importance (greater weight) to audio stimulation when evaluating valence.
(C) Participants integrate the two modalities according to an algebraic rule.

### Studied Conditions and Stimuli

#### Unimodal Conditions

*Audio stimuli* (Φ*AS*) consisted of samples of acted speech. The methodology used for collecting the speech signals was based on the GEMEP corpus (Bänziger et al., 2012). The digital samples (32 bit, mono, 44.1 kHz sampling rate) were recorded with a male adult speaker uttering a pseudo speech sentence (“nekal ibam soud molen!”). To perform this utterance, the speaker was provided with a list of short illustrative descriptions of the meaning of the emotion term and three scenarios for each of three emotion categories. The studied emotions consisted of anger (negative EV), joy (positive EV) and neutrality (no EV). These three categories enable us to study the perception of positive vs negative emotions. After the voices were recorded in the studio of the laboratory, we selected one sample for each of the two emotions, as well as neutrality. Then, the three selected samples were normalized for power (RMS). The expressions ranged from 1200 to 1400 ms duration. To obtain intermediate stimuli levels, voice morphing was performed using the STRAIGHT programmed in Matlab (MathWorks) between the neutral expression and the two emotional expressions. STRAIGHT (Kawahara and Irino, 2005; Kawahara and Morise, 2011) performs an instantaneous pitch-adaptive spectral smoothing in each stimulus for separation of contributions to the voice signal arising from the glottal source (including F0) versus supralaryngeal filtering (distribution of spectral peaks, including the first formant, F1). For example, STRAIGHT has been used to show that averaging voices with auditory morphing increases vocal attractiveness (Bruckert et al., 2010). We manually identified time-frequency landmarks in each stimulus to be put in correspondence across the neutral and anger voices, and then across the neutral and joy voices. Morphed stimuli were then generated by resynthesizing based on the interpolation (linear for time; logarithmic for F0, frequency, and amplitude) of these time–frequency landmark templates. Finally, we obtained five voice expressions, ranging from anger (maximum negative valence = Φ*AS*_-100%_) to joy (maximum positive valence = Φ*AS*_+100%_), corresponding to Φ*AS*_-100%_, Φ*AS*_-50%_, Φ*AS*_0%_, Φ*AS*_+50%_, and Φ*AS*_+100%_. We conducted a preliminary experiment to examine how the generated voice stimuli were perceived among a group of 15 adult participants. Each stimulus was repeated 6 times and presented in a random order. After each stimulus, participants were asked to evaluate the emotion valence using a track-bar ranging from very positive to very negative. We performed a repeated-measures analysis of variance (ANOVA) on participants’ mean rating, with a significance threshold set at *p* < 0.05. We found a significant difference between the levels of voice stimuli [*F*(4,56) = 88.93, ε = 0.40, *p* < 0.001, and η^2^*_p_* = 0.86]. *Post hoc* tests (with a Bonferroni corrected *p* < 0.05/10) revealed that each level was significantly different from each other level. More precisely, valence ratings increased linearly with audio levels [*F*(1,14) = 117.50, *p* < 0.001]. This linear trend, showed that perceived EV follows qualitatively and proportionally the morphing continuum of the Φ*AS* (EV = 0.58 ^∗^ Φ*AS* – 0.14, and *R*^2^ = 0.98).

*Tactile stimuli* (Φ*TS*) consisted of air jet tactile stimulation applied on the forearm. The controlled parameters of the tactile stimulation were i) the levels of air jet flow rate, and ii) the levels of movement speed of the rotating nozzle. Three types of tactile stimuli were presented to participants: (1) a tactile stimulus of a high intensity level (Φ*TS_HI_*), corresponding to a high flow rate (50 nl/min corresponding to blowing force of 0.682N) together with a high movement velocity (12 rad/s), (2) a tactile stimulus of a medium intensity level (Φ*TS_ME_*), corresponding to a medium flow rate (25 nl/min corresponding to blowing force of 0.341) with a null movement velocity, and (3) a tactile stimulus of a low intensity level (Φ*TS_LO_*), corresponding to a low flow rate (7.5 nl/min corresponding to blowing force of 0.172N) with a slow movement velocity (0.6 rad/s). These parameters of the air jet were chosen according to a previous study that related the air jet tactile features and the perceived valence (Tsalamlal et al., 2013). This previous study (Tsalamlal et al., 2013) showed that Φ*TS_HI_* was perceived with a negative valence, Φ*TS_ME_* was rated as neutral, and Φ*TS_LO_* was rated positively. The number of levels of tactile stimuli (3 levels) is different from the number levels of audio stimuli (5 levels) because intermediate tactile levels were not clearly discriminated by participants.

#### Bimodal Condition

*Audio-tactile stimuli* (Φ*ASTS*) consisted of *audio stimuli* (Φ*AS*) presented simultaneously with *tactile stimuli* (Φ*TS*). Each audio stimulus type was combined with all of the tactile stimulus types: five audio expressions ^∗^ three tactile levels.

### Measures

**FIGURE 3:**
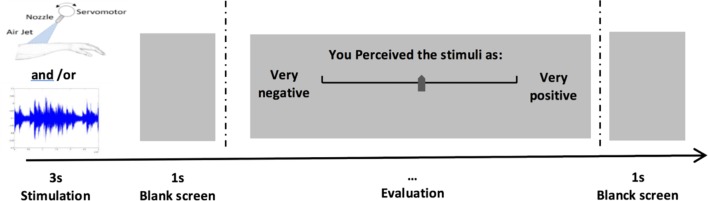
Experimental protocol. Participants perceived stimuli during 3s, then they evaluated Ev on a continuous scale with a track bar.

After each stimulus, participants evaluated EV on a continuous scale (a track bar) ranging from very negative (-100) to very positive (+100) (see **Figure 3**).

### Procedure

The participants were seated in front of a desk with the tactile device, a screen, and a computer mouse. Then, the headphone was set. A practice session of six stimuli was completed to ensure that participants understood the course of the experiment. In this session, the extreme values of the different stimuli were displayed. Participants had to maintain their left forearm inside the box where the tactile device was produced (**Figure 2**). Participants were instructed to keep the same body position during the whole experiment.

The experiment comprised six blocks of stimuli. The unimodal conditions consisted of 30 Φ*AS stimuli* (five levels ^∗^ six repetitions) and 18 Φ*TS stimuli* (three levels ^∗^ six repetitions). The bimodal conditions consisted of 90 Φ*ASTS stimuli* (five audio ^∗^ three haptic ^∗^ six repetitions). Participants performed a total of 138 trials (without including the practice session trials). Each trial lasted 3 s. In bimodal stimuli, the tactile stimulation lasted 3 s, whereas the audio stream started 1 s after the tactile stimuli onset, with a duration ranging from 1200 to 1400 ms. The sequence of bimodal and unimodal blocks was ordered randomly across participants. Participants used their right arm to indicate their responses with the mouse. After each stimulus, participants moved the track bar on the screen with the mouse to provide their rating of EV.

## Results

Functional measurement analyses were performed to identify the integration process used by participants to combine Φ*AS* and Φ*TS*. This approach was based on both the visual inspection of graphical patterns (called integration graphs) and statistical analyses. First, we highlighted the average response of the group of participants. Then, we examined the responses on an individual level to the different bimodal combination strategies displayed by each participant.

### Global Analyses

#### Integration Graphs

**FIGURE 4:**
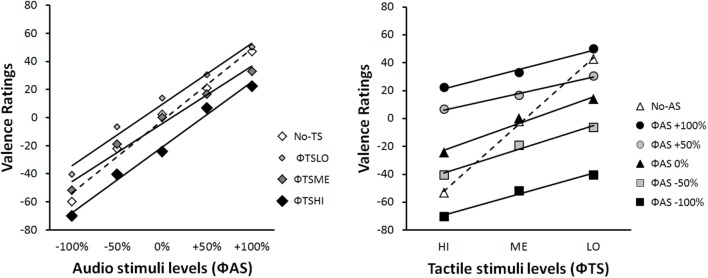
Integration graphs of mean participants’ EV responses expressed as a function of the physical scale (Φ) for each stimulus category.

**FIGURE 5:**
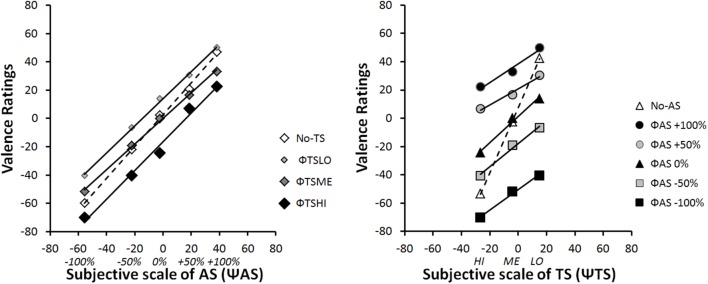
Integration graphs of mean participants’ EV responses expressed as a function of their subjective (Ψ) scaling for each stimulus category. Ψ-values correspond to marginal mean of EV ratings according each Φ level.

**Figure 4** provides a classical illustration of the data. The mean EV rating is expressed as a function of each stimulus category in terms of physical scale (Φ). In contrast, **Figure 5** illustrates the integration graph of the same data, where the mean EV rating is expressed as a function of the subjective valence scaling (Ψ*AS*) of the audio-physical stimuli (Φ*AS*) and of the different levels of tactile stimuli Φ*TS*. Subjective values (Ψ) are approximated by the marginal means of the responses given by participants for each physical Φ condition (Anderson, 1996), p.73). Accordingly, each coordinate along the Ψ*AS*_-100%_ to Ψ*AS*_+100%_ abscissa of **Figure 5** is the functional estimate of the physical stimulus values ranging from Φ*AS*_-100%_ to Φ*AS*_+100%_ in **Figure 4**. For example, for extreme negative audio stimuli (Φ*AS*_-100%_), participants estimated EV as follows: -60 when presented alone (Φ *no-TS*), -70 when presented with high intensity tactile stimulation (Φ*TS_HI_*), -52 with medium intensity tactile stimulation (Φ*TS_ME_*) and -40.4 with low intensity tactile stimulation (Φ*TS_LO_*) (see **Figure 4**: Left panel). Accordingly, the corresponding subjective value of Ψ*AS*_-100%_ (i.e., Ψ*min*) was -55, computed as the marginal mean of -60, -40.4, -51.8, and -70 (see **Figure 5**: Left panel). Data and computation of all our factorial plots are available from https://1drv.ms/f/s!Ar7_iO4FFoZMmliQTjv-OejI6K91.

#### Statistical Analyses

To support observations made using integration graphs, the data were analyzed using classical statistics. We performed ANOVAs followed by *post hoc* pairwise (Bonferroni corrected) comparisons to study the effect of each experimental factor (audio and tactile stimuli) on the perception of valence. The significance threshold was set at *p* < 0.05. Moreover, we report effect size measures such as η^2^*_p_* for ANOVAs, *R*^2^ for linear trends, and Cohen’s d (with 95% confidence interval) for differences between independent or paired samples. Finally, in order to reduce Type I errors (due to violations of the sphericity assumption), we applied Greenhouse-Geisser corrections to the ANOVA degrees of freedom, and provide the corresponding epsilon value.

##### Unimodal stimuli

Separate repeated-measures ANOVAs were performed on the participants’ EV responses for the audio and tactile unimodal blocks. The following within-subjects’ factors were considered for analysis: unimodal *TS* with three levels (Φ*TS_HI_*, Φ*TS_ME_*, and Φ*TS_LO_*) and unimodal *AS* with five levels (Φ*AS*_-100%_, Φ*AS*_-50%_, Φ*AS*_0%_, Φ*AS*_+50%_, and Φ*AS*_+100%_). For unimodal *TS*, the results showed significant differences for EV between the three different *TS* [*F*(2,44) = 101.41, ε = 0.64, *p* < 0.001, and η^2^*_p_* = 0.82]. Φ*TS_HI_* were perceived as negative (i.e., unpleasant) (*M* = -53.45, *SD* = 30.35); ΦTS*_ME_* were perceived as neutral (*M* = -2.23, *SD* = 12.54); and Φ*TS_LO_* were perceived as positive (i.e., pleasant) (*M* = 42.60, *SD* = 18.61). *Post hoc* analyses (with Bonferroni corrected alpha = 0.05/3) revealed that all pairwise comparisons were statistically significant (all *p*s < 0.001). These results are consistent with the above-mentioned hypothesis, H1.A.

For unimodal Φ*AS*, the results showed a main effect of *AS* [*F*(4,88) = 123.48, ε = 0.38, *p* < 0.001, and η^2^*_p_* = 0.85] due to a significant linear increase in EV with the five different levels of *AS* [*F*(1,22) = 161.94, *p* < 0.001, and *R*^2^ = 0.98]. *Post hoc* tests (with Bonferroni corrected alpha = 0.05/10) indicated that all pairwise comparisons of EV ratings for the different intensities of audio stimulation were statistically significantly different from each other (all *p*s < 0.001). These results are consistent with the above-mentioned hypothesis, H1.B., and the preliminary study (see section “Studied Conditions and Stimuli”).

Functional measurement plots offer direct access to the internal scale range corresponding to the valuation process of IIT. This scale range (i.e., the difference between Ψmin and Ψmax) quantifies the effect size of each source of information (*AS* and *TS*) on the estimation of the EV of the multimodal stimulus. **Figure 5** illustrates the fact that the overall effect of *AS* (Ψ*AS*_+100%_ minus Ψ*AS*_-100%_) on EV was much greater than that the effect of *TS* (Ψ*TS_HI_* – Ψ*TS_LO_*). Due to individual differences in the modality effect, the relative importance of each modality when making EV judgments will be examined in the next section (see section “Individual Analyses”).

##### Bimodal stimuli

The pattern in the integration graph is a direct picture of the experimental effects. Therefore, the rule by which two modalities are integrated to form the one EV response can be diagnosed from those factorial plots (Anderson, 1996). The interaction patterns displayed in **Figures 4**, **5** are consistent with an averaging rule observed for combining both *AS* and *TS*. According to IIT, averaging is suggested by parallel lines for bimodal conditions, together with a crossover line for the unimodal condition (Anderson, 1982, 1996; Zalinski and Anderson, 1989). Parallelism pattern supports an adding-type rule (whether adding or averaging), as if the participant assigns values to each modality and adds them to determine EV. Under an adding hypothesis, the information added by a given modality should have the same directional effect at all the line points. To paraphrase (Zalinski and Anderson, 1989; Anderson, 1996), the solid lines of **Figures 4**, **5** would lie above the dashed line, if the added information was positive; whereas the solid lines would lie above the dashed line, if the added information was negative. Therefore, the adding hypothesis does not account for the crossover of the dashed line.

However, a straightforward account of the crossover is provided by the averaging hypothesis. The dashed lines of **Figures 4**, **5** indicate that information of each modality alone (unimodal stimuli) is near neutral in net value, corresponding to the midpoint of the response scale (i.e., close to EV = 0). Therefore, if this information was averaged, the points of the solid lines would be pulled in toward the center of the graph. As a consequence, it would average up the low levels of each modality and average down the high levels, thereby creating the dashed line crossover (Zalinski and Anderson, 1989; Anderson, 1996).

In order to provide a first statistical support to the averaging hypothesis, we performed two-way repeated-measures ANOVAs on participants’ ratings of the stimuli, with two within-subjects’ factors (*TS* and *AS*) considered for the analysis. However, the number of levels of each factor varied with the interaction graph under examination. The ANOVA on data from **Figure 4** Left panel considered *AS* with five levels (-100%, -50%, 0%,+50%, and +100%), and *TS* with four levels (HI, ME, LO, and *no-TS*). The results showed a significant main effect of *AS* [*F*(4,88) = 107.51, ε = 0.39, *p* < 0.001, and η^2^_p_ = 0.83] and *TS* [*F*(3,66) = 31.34, ε = 0.60, *p* < 0.001, and η^2^_p_ = 0.59], and an interaction between both factors [*F*(12, 264) = 4, ε = 0.34, *p* < 0.001, and η^2^_p_ = 0.15] on the perceived EV. The ANOVA on data from **Figure 4** Right panel considered TS with three levels (HI, ME, and LO), and *AS* with six levels (-100%, -50%, 0%,+50%,+100%*_,_* and *no-AS*). The results showed a significant main effect of *TS* [*F*(2,44) = 75.38, ε = 0.66, *p* < 0.001, and η^2^_p_ = 0.77] and *AS* [*F*(5,110) = 83.76, ε = 0.36, *p* < 0.001, and η^2^_p_ = 0.79], and an interaction between both factors [*F*(10,120) = 23.57, ε = 0.35, *p* < 0.001, and η^2^_p_ = 0.52] on the perceived EV. Finally, in order to test the parallelism hypothesis, a last ANOVA considered *AS* with five levels (from -100% to+100%) and TS with three levels (HI, ME, and LO), i.e., without the *no-AS* and the *no-TS* levels. The results showed that the main effects remained significant [*TS*: *F*(2,44) = 38.20, ε = 0.65, *p* < 0.001, and η^2^_p_ = 0.63; *AS*: *F*(4,88) = 91.68, ε = 0.39, *p* < 0.001, and η^2^_p_ = 0.81], and that the interaction turned out non-significant [*F*(8,176) = 2.51, ε = 0.38, *p* = 0.068, n.s., and η^2^_p_ = 0.10], thereby supporting the parallelism of the bimodal lines in **Figures 4**, **5**.

#### Discussion

For unimodal conditions, participants were able to discriminate between different levels of EV according to the various levels of AS and TS. The validation of H1.A suggests that the air jet stimulation communicates different levels of valence. The controlled stimulation parameters of the air jet stimulation (i.e., air flow rate and nozzle movement velocity) influence participant’s perception of EV. This result is consistent with our previous study (Tsalamlal et al., 2013). The validation of H1.B suggests that the morphing operation of voice was efficient since it produces distinct intermediate levels of EV. Based on the literature, the discrimination of natural prosodic voice stimuli was predictable (Bänziger et al., 2009, 2012). However, our ability to discriminate morphed levels was not obvious *a priori*.

Integration graphs, together with the statistical analyses, highlight the fact that participants based their judgment on both voice expression and tactile stimulation to evaluate EV. The overall pattern of factorial plots suggests that the main integration rule was averaging. Moreover, the results showed that the internal (subjective) scale for voice expression was larger than was the tactile stimulation scale. This difference may be explained by the quality of the expressions. The voice expressions that we designed are close to natural expressions (except that they did not correspond to syntactically and semantically correct sentences), whereas air jet stimulation might be perceived as being quite different from everyday haptic stimulation. Still, our results suggest that air jet stimulation provides an effective means of mediating emotion communication.

### Individual Analyses

The global analyses described above suggest that participants used an averaging rule to combine incoming information from both AS and TS. However, the global averaging integration of the two modalities might result in different integration rules at the individual level. We examined factorial plots and performed ANOVAs at the individual level to study individual differences in the multimodal integration process. First, we performed a clustering operation to classify participants into different groups that display similar integration patterns within-group, but also include differences between groups. Then, we conducted functional measurement analyses on each group to examine its integration process. All the program and data files are available from https://1drv.ms/f/s!Ar7_iO4FFoZMmliQTjv-OejI6K91, together with an Appendix explaining how the results were obtained.

#### Cluster Analysis

To examine if different participants displayed different modes of integration (tested algebraic rules: adding, averaging and multiplying), we performed a clustering analysis on the group of participants on the basis of their response patterns. In the context of IIT, previous research (Hofmans and Mullet, 2013) proposed the use of an agglomerative hierarchical clustering procedure, together with the centroid agglomerative algorithm (distance between two clusters is defined as the difference between the centroids, i.e., the cluster averages). This algorithm includes all data points and is less affected by outliers than are other hierarchical methods. After visual inspection of the individual graphical analysis, we opted for a three-cluster solution. Two clusters contained more than one participant. Cluster 1 included 17 participants, cluster 2 included five participants.

#### Integration Graphs

**FIGURE 6:**
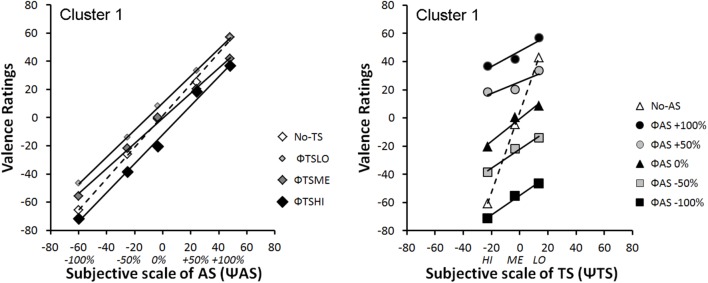
Mean integration graphs of Cluster 1 participants as a function of their subjective (Ψ) scaling for each stimulus category.

**FIGURE 7:**
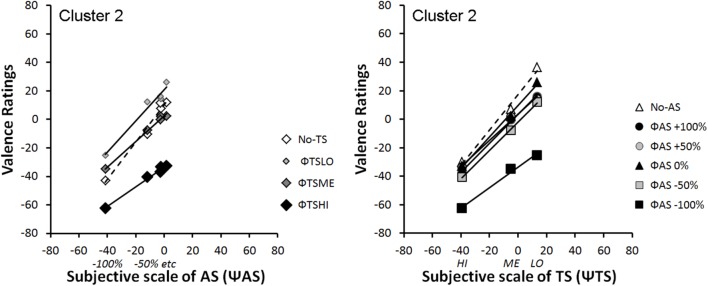
Mean integration graphs of Cluster 2 participants as a function of their subjective (Ψ) scaling for each stimulus category.

**Figures 6**, **7** represent integration graphs of participants’ mean EV from Clusters 1 and 2, respectively, as a function of their subjective (Ψ) scaling for each stimulus category. For the sake of simplicity, we did not display participants’ mean EV as a function of the physical scale (Φ) for each stimulus category. The factorial patterns of the first cluster closely resemble those of **Figure 4**, with near-parallel lines for bimodal rating, and a crossover dashed line for the unimodal condition (see **Figure 6**). Cluster 1 included 80% of the participants. The second cluster shows different response patterns (see **Figure 7**). In this cluster, the positive levels of *AS* stimuli within each *TS* level are not well discriminated, as illustrated in **Figure 7** Left panel by the bundled points along the *TS* lines, close to the midpoint of the response scale. Moreover, in contrast to Cluster 1, the subjective scaling range for each modality (Ψmax minus Ψmin) is rather similar between *AS* and *TS* (just slightly greater for *TS*). Although the pattern displayed by Cluster 1 is a signature of an averaging integration rule, this is less obvious for Cluster 2, especially from visual inspection of **Figure 7** Right panel showing no crossover line for unimodal *TS* stimuli (no-*AS* level). To support the integration rules deduced from the graphical patterns, we conducted ANOVAs on each cluster, with a significance threshold set at *p* < 0.05.

#### Statistical Analyses

We followed the same logic as the statistical analyses for bimodal stimuli on the overall data.

**Cluster 1:** the ANOVA on the data displayed as a function Ψ*AS* (see **Figure 6**: Left panel) revealed main effects of *AS* [*F*(4,64) = 190.71, ε = 0.64, *p* < 0.001, and η^2^_p_ = 0.92] and *TS* [*F*(3,48) = 22.16, ε = 0.69, *p* < 0.001, and η^2^_p_ = 0.58], as well as an interaction between both factors on perceived valence [*F*(12, 192) = 3.68, ε = 0.32, *p* = 0.011, and η^2^_p_ = 0.19]. The ANOVA on the EV data displayed as a function Ψ*TS* (see **Figure 6**: Right panel) revealed a significant main effect of *TS* [*F*(2,32) = 87.09, ε = 0.59, *p* < 0.001, and η^2^_p_ = 0.85] and *AS* [*F*(5,80) = 155.04, ε = 0.59, *p* < 0.001, and η^2^_p_ = 0.91], and an interaction between both factors [*F*(10, 160) = 31.25, ε = 0.41, *p* < 0.001, and η^2^_p_ = 0.66]. Finally, in order to test the parallelism hypothesis, a last ANOVA considered all the levels of both factors (*AS* and *TS*) but the unimodal stimuli (*no-AS* and *no-TS*). The results showed that the main effects remained significant [*TS*: *F*(2,32) = 34.76, ε = 0.58, *p* < 0.001, and η^2^_p_ = 0.68; *AS*: *F*(4,64) = 160.92, ε = 0.68, *p* < 0.001, and η^2^_p_ = 0.91], and that the interaction turned out non-significant [*F*(8,128) = 2.38, ε = 0.34, *p* = 0.088, n.s., and η^2^_p_ = 0.13], thereby supporting the near-parallelism of the bimodal lines in **Figure 6**.

**Cluster 2:** the ANOVA on the data displayed as a function Ψ*AS* (see **Figure 7**: Left panel) revealed main effects of the two factors *AS* [*F*(4,16) = 50.95, ε = 0.63, *p* < 0.0001, and η^2^_p_ = 0.93] and *TS* [*F*(3,12) = 16.35, ε = 0.42, *p* = 0.008, and η^2^_p_ = 0.80], as well as a non-significant interaction between both factors on perceived valence [*F*(12,48) = 2.97, ε = 0.19, and *p* = 0.097, n.s.] although its effect size remained substantial (η^2^_p_ = 0.43). The ANOVA on the data displayed as a function Ψ*TS* (see **Figure 7**: Right panel) revealed a significant main effect of *TS* [*F*(2,8) = 13.41, ε = 0.55, *p* = 0.018, and η^2^_p_ = 0.77] and *AS* [*F*(5,20) = 28.63, ε = 0.55, *p* < 0.001, and η^2^_p_ = 0.88], and a non-significant interaction between both factors [*F*(10,40) = 1.07, ε = 0.18, *p* = 0.38, n.s., and η^2^_p_ = 0.21]. Finally, in order to test the parallelism hypothesis, a last ANOVA considered all the levels of both factors (*AS* and *TS*) but the unimodal stimuli. The results showed that the main effects remained significant [*TS*: *F*(2,8) = 16.91, ε = 0.59, *p* < 0.01, and η^2^_p_ = 0.81; *AS*: *F*(4,16) = 53.62, ε = 0.63, *p* < 0.001, and η^2^_p_ = 0.93], and that the interaction remained non-significant [*F*(8,32) = 2.06, ε = 0.26, *p* = 0.186, n.s., and η^2^_p_ = 0.34], thereby supporting the near-parallelism of the bimodal lines in **Figure 7**.

Integration graphs, together with the statistical analyses, indicate that the two groups of participants based their judgment on both voice expressions and tactile stimulations to evaluate EV. However, while there is clear evidence that participants from Cluster 1 integrated both sources of information using an averaging rule, it is less clear cut for Cluster 2 besides the *no-TS* crossover line for the data displayed as a function Ψ*AS* (see **Figure 7**: Left panel). Therefore, we evaluated the goodness of fit of the averaging model to the data using dedicated software.

#### Model Fitting

The results showed that the integrated response Ψ*R* is a consequence of an averaging rule type (Equation 1). The averaging model states that Ψ*R* is a weighted sum of single modality values divided by the sum of the weights. If the levels of a factor do not have the same weight, then this factor is called Differentially Weighted and the integration rule becomes non-linear. If all of the levels of a factor have the same weight (within *AS* or *TS*), then this factor is called Equally Weighted. However, these weights do not need to be the same for each factor. The sum of weights in the denominator has the same value in each cell of the design and can be absorbed into an arbitrary scale unit. Accordingly, this model has a linear form.

$$\Psi R=\frac{w_{0}\Psi s_{0}+\Sigma w_{i}\Psi s_{i}}{w_{0}+\Sigma w_{i}}$$

Where:

Ψ*_Si_*: are the scale values of a single stimulus variable.

*ω_i_*: denotes the weight of each value.

The initial state of the process is represented by ω_0_ and Ψ_*S*0_. The initial state enables the model to take into account the set-size effect in which pieces of added information of equal values can produce a more extreme response. After the identification of the averaging rule based on functional measurement, it was necessary to verify that the model presented a good fit to the data. We used the R-Average software program (Vidotto et al., 2010), which independently estimates the weight and scale values of the stimuli. Estimation was performed on a single-subject basis, resting on the equal weight averaging model (EAM). Goodness-of-fit was evaluated using separate repeated measures ANOVAs over the residuals left by the model tested for each cluster:

• **Cluster 1:** The ANOVA on the residuals of the data displayed as a function Ψ*AS* (see **Figure 6**: Left panel) revealed that the main effects of *TS* and *AS* were canceled out (respectively, *F*(3,48) < 1, n.s., and *F*(4,64) < 1, n.s.), as well as the *TS* ^∗^ *AS* interaction [*F*(12,192) = 2.06, ε = 0.32, *p* = 0.10, n.s., and η^2^_p_ = 0.11]. Similarly, the ANOVA on the residuals of the data displayed as a function Ψ*TS* (see **Figure 6** Right panel) revealed that the main effects of *TS* and *AS* were canceled out (respectively, *F*(2,32) < 1, n.s., and *F*(5,80) < 1, n.s.), as well as the *TS* ^∗^ *AS* interaction [*F*(10,160) = 1.54, ε = 0.41, *p* = 0.13, n.s., and η^2^_p_ = 0.09].
• **Cluster 2:** The ANOVA on the residuals of the data displayed as a function Ψ*AS* (see **Figure 7**: Left panel) revealed that the main effects of *TS* and *AS* were canceled out in the residuals (respectively, *F*(3,12) < 1, n.s., and *F*(4,16) < 1, n.s.), as well as the *TS* ^∗^ *AS* interaction [*F*(12,48) = 1.67, ε = 0.19, *p* = 0.24, n.s., and η^2^_p_ = 0.29]. Similarly, The ANOVA on the residuals of the data displayed as a function Ψ*TS* (see **Figure 7**: Right panel) revealed that the main effects of *TS* and *AS* were canceled out (respectively, *F*(2,8) < 1, n.s., and *F*(5,20) < 1, n.s.), as well as the *TS* ^∗^ *AS* interaction [*F*(10,40) < 1, n.s.].

These results are consistent with our hypothesis H2.C that participants integrated both modalities according to an algebraic rule, namely the equal weight averaging rule.

#### Measure of Importance of the Modalities

**FIGURE 8:**
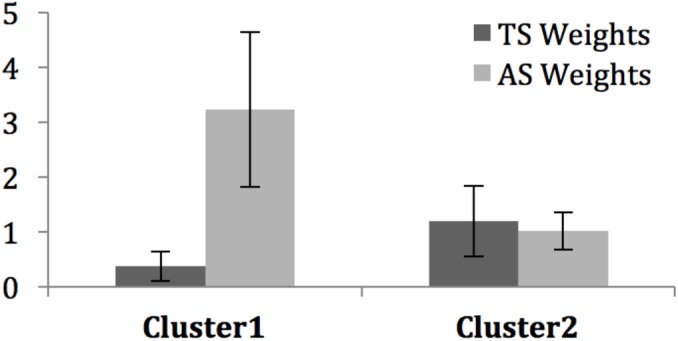
Mean individual weights (±SD) for TS and AS in the averaging model fit of each cluster.

**Figure 8** presents the average estimated weights. A first analysis showed that each of the four mean values was significantly different from zero, whether for Cluster 1 [*TS*: *t*(16) = 5.61, *p* < 0.001, Cohen’s *d* = 1.36 with 95%CI = (0.68, 2.02); *AS*: *t*(16) = 9.47, *p* < 0.001, Cohen’s *d* = 2.30 with 95%CI = (1.38, 3.21)], or for Cluster 2 [*TS*: *t*(4) = 2.55, *p* = 0.021, Cohen’s *d* = 1.64 with 95%CI = (0.21, 3.01); *AS*: *t*(4) = 3.37, *p* = 0.0039, Cohen’s *d* = 2.68 with 95%CI = (0.69, 4.67)]. Given the robustness of these differences (all *ds* > 0.80), we can confidently assume that participants based their judgments on both modalities to evaluate EV, thereby validating our hypothesis H2.A. Moreover, none of Cohen’s *d* confidence intervals included 0.20, suggesting that the contribution of each modality is not small in the parent population.

Weights are obtained on a ratio scale (Anderson, 1982), thus allowing for direct comparisons within the groups. For the participants from Cluster 1, the weight of voice stimuli was higher than the weight of the tactile stimuli [*t*(16) = 7.42, *p* < 0.001, Cohen’s *d* = 1.80 with 95%CI = (1.01, 2.57)]. For Cluster 2, the weights of the voice and tactile stimuli were not significantly different [*t*(4) = 0.36, *p* = 0.74, n.s.]. However, the non-significant finding in Cluster 2 does not exclude the possibility that a difference might exist in the parent population that was not detectable given the small number of participants in Cluster 2. The observed effect in Cluster 2 is a slightly greater weight in favor to tactile stimuli (0.17 unit of weight) over voice stimulation. In comparison, Cluster 1 shows a 18 times greater difference (2.89 units of weight), in favor of voice stimulation, along the lines of hypothesis H2.B. Therefore, we conducted fiducial Bayesian analyses (Lecoutre and Poitevineau, 2014) on the data of Cluster 2 in order to estimate in the parent population the magnitude of the effect, if any. Results indicated that there is a 95% probability (or guarantee) that, in the parent population, the effect observed in Cluster 2 is smaller than 1.35 unit of weight in absolute value, i.e., whether tactile>voice, or voice>tactile. In order words, if there is a greater weight in favor to tactile stimuli over voice stimulation in the parent population, at best, it will be about 8 times greater than what we observed in Cluster 2. In contrast, fiducial Bayesian analyses on the data of Cluster 1 showed that there is a 95% guarantee that, in the parent population, the effect observed in Cluster 1 is greater than 2.21 units of weight. In other words, if there are individuals in the parent population giving more weight to tactile than voice stimulation, this effect will be much smaller in magnitude than that for voice over tactile stimulation.

To make way for comparisons across groups, the values were normalized per subject to their total sum (including w0, a weight parameter of the averaging model not reported here). Values represent the average relative importance (varying between 0 and 1) of each level in each factor. Overall, touch had a greater importance for Cluster 2 than for Cluster 1 [*t*(20) = 3.46, *p* = 0.003, Cohen’s *d* = 1.76 with 95%CI = (0.61, 2.88)]. Overall, the importance of voice was greater for Cluster 1 than for Cluster 2 [*t*(20) = 5.40, *p* < 0.001, Cohen’s *d* = 2.75 with 95%CI = (1.42, 4.03)].

Finally, all the significant differences reported in this subsection passed the Bonferroni corrected alpha = 0.05/8 = 0.0063, but the mean TS weight for Cluster 2 compared to zero. However, the fact that its Cohen’s *d* confidence intervals did not include 0.20 suggests that the contribution of this modality cannot be deemed negligible in the parent population.

#### Discussion

Both the visual inspection of factorial plots and the clustering analysis enabled to identify the two groups of participants that accounted for all the individual integration patterns, except for one participant. The first group was composed of 4/5 of the participants, whereas the second group involved 1/5 of the participants. Functional analyses showed that these groups presented different types of averaging patterns (**Figure 8**). A dedicated model fitting software showed that EAM was the best fitted model for both groups. Further analysis of the residuals left by the model confirmed the goodness of fit. For each group, the weights of each audio and tactile modality were calculated. The two differed in the evolution of EV according to *AS* and *TS*. The first group gave higher importance to the audio modality when combining voice expression and tactile stimulation, whereas the second group gave similar importance to both modalities.

## General Discussion

Most studies about multimodal perception have focused on the investigation of audio and visual signals. The haptic cues have received less attention. This lack of interest may be due to two main factors. First, the design of haptic stimulation suitable for emotion communication is very challenging. Second, there are methodological obstacles in the study of touch such as the difficulty to observe and analyze the social haptic interaction (ex., intrusiveness of sensors), and the difficulty to elicit spontaneous affective haptic behavior during classical experiments (i.e., controlled experiment, adherence to the context). Furthermore, most studies that address touch affective communication have considered this modality alone.

We proposed the use of an air jet tactile stimulation for conveying affective touch stimuli. This stimulation strategy does not require any physical contact between the user and mechanical structures. It also permits the generation of low amplitude forces that might be suitable to communicate affective features.

The present paper presented an experimental study that aimed at highlighting how air jet tactile stimulation is combined with audio cues when estimating EV. Three types of stimuli were presented to participants (i.e., vocal expressions of emotion, tactile stimulations, and combined voice and tactile stimuli). The responses of participants were analyzed using functional measurements. The results revealed that both groups of participants effectively discriminated EV from audio and tactile stimuli.

For unimodal tactile stimulation, low intensity *TS* were perceived more positively than medium and high intensity stimuli. High intensity *TS* were perceived more negatively than medium and low intensity stimuli. These results are consistent with previous research investigating the relationships between tactile physical features and the evaluation of valence (Tsalamlal et al., 2013). Along the same lines, researchers have demonstrated the existence of nerve endings located in the skin that effectively convey the sensory signal to the hedonic nervous system that processes the feelings of pleasure directly produce hedonic values (Hertenstein, 2002). Regarding unimodal audio stimulation, the anger expression was rated negatively, and expressions of joy were rated positively. Generally, the rating of valence was proportional to the morphing level of the expression. These results underline the role of vocal expression in communicating emotions (Juslin and Laukka, 2003). The STRAIGHT program was definitely a useful tool for performing vocal morphing to produce intermediate emotional levels.

Regarding bimodal stimuli, the functional measurement approach revealed that participants combined voice and touch stimulations with an equal weight averaging rule. Cluster analyses revealed an averaging pattern. Most of the participants (80%) presented linear evolution of EV ratings according to the audio stimuli. Some participants presented curved evolution of EV ratings according to the audio stimuli. The first group showed a predominance of the audio channel over the tactile stimulation for estimating EV. The second group gave similar importance to the two modalities since not all audio stimuli were discriminated. Future research should address several issues. Integration of audio-tactile expressions coming from the same source (e.g., using haptic expressions of emotion generated by users (Gaffary et al., 2013; Bailenson et al., 2007)) should be investigated. In addition, studies should highlight individual differences in the integration of the different modalities over the course of EV perception. Finally, the effects of gender, age, and personality traits on sensitivity to social touch and social voice (Kreiman et al., 1992; Gallace and Spence, 2010) should be examined.

## Conclusion

Considering all the results of our study, we conclude that touch conveyed by haptic devices should play a major role in affective communication. Even if our tactile stimulation technique (i.e., tactile stimulation using air jet) is unusual, our results revealed that it could effectively communicate EV. Using two channels simultaneously changed the perception of emotions compared to a unimodal channel of communication. Obviously, in multimodal social interactions the interpretation of affect is modulated by the context in which the stimulations (whether visual, audio, and/or tactile) take place, and the familiarity or relationship with person, or artificial agent, may it be a virtual human (Oker et al., 2015) or humanoid robot (Artstein et al., 2016). Therefore, future research is needed to evaluate to what extent context might override subtle differences in stimulus qualities.

This study highlighted the usefulness of formal algebraic models for representing the processes underlying multimodal emotional information integration. Such results might be useful for designing and integrating tactile stimulation in multimodal emotional communication platforms (for example for long distance mediated communication or an expressive virtual agent (Courgeon and Clavel, 2013). Our study may also contribute to the design a computational model that will allow for automatic recognition and display of affective cues through touch simultaneously conveyed with other modalities.

## Ethics Statement

Research ethics committee of Université Paris-Saclay.

## Author Contributions

YT contributed to global theoretical framework, implementation of the platform, user study, data analysis, state of the art. M-AA contributed to theoretical framework on Information Integration Theory (IIT), data modeling with IIT, discussion of results, state of the art. J-CM contributed to affective computing background, recommendation of study design (protocol, measures, audio stimuli design, etc.) state of the art. MA contributed to haptic perception background, recommendations for study design (hardware recommendations, tactile stimuli design, etc.) state of the art.

## Conflict of Interest Statement

The authors declare that the research was conducted in the absence of any commercial or financial relationships that could be construed as a potential conflict of interest.

## References

[B1] Ackerley R. Eriksson E. Wessberg J. (2013). Ultra-late EEG potential evoked by preferential activation of unmyelinated tactile afferents in human hairy skin. *Neurosci. Lett.* 535 62–66. 10.1016/j.neulet.2013.01.004 23328438

[B2] Adolphs R. (2002). Recognizing emotion from facial expressions: psychological and neurological mechanisms. *Behav. Cogn. Neurosci. Rev.* 1 21–62. 10.1177/1534582302001001003 17715585

[B3] Alhalabi Osama M. Susumu S. (2001). Tele-handshake: a cooperative shared haptic virtual environment. *Eurohaptics* 2001 60–64.

[B4] Ammi M. Demulier V. Caillou S. Gaffary Y. Tsalamlal Y. Martin J.-C. (2015). “Haptic human-robot affective interaction in a handshaking social protocol,” in *Proceedings of the IEEE Human-Robot Interaction Conference*, Portland, OR.

[B5] Anderson H. (1996). *A Functional Theory of Cognition.* London: Psychology Press.

[B6] Anderson N. H. (1971). Integration theory and attitude change. *Psychol. Rev.* 78 171–206. 10.1037/h0030834

[B7] Anderson N. H. (1981). *Foundations of Information Integration Theory.* New York, NY: Academic Press.

[B8] Anderson N. H. (1982). *Methods of Information Integration Theory.* New York: Academic Press.

[B9] App B. McIntosh D. N. Reed C. L. Hertenstein M. J. (2011). Nonverbal channel use in communication of emotion: how may depend on why. *Emotion* 11 603–617. 10.1037/a0023164 21668111

[B10] Artstein R. Traum D. Boberg J. Gainer A. Gratch J. Johnson E. (2016). “Niki and Julie: a robot and virtual human for studying multimodal social interaction,” in *Proceedings of the 18th ACM International Conference on Multimodal Interaction - ICMI 2016*, Boulder.

[B11] Avraham G. Nisky I. Fernandes H. L. Acuna D. E. Kording K. P. Loeb G. E. (2012). Toward perceiving robots as humans: three handshake models face the turing-like handshake test. *IEEE Trans. Haptics* 5 196–207. 10.1109/TOH.2012.16 26964106

[B12] Bailenson J. N. Yee N. Brave S. Merget D. Koslow D. (2007). Virtual interpersonal touch: expressing and recognizing emotions through haptic devices. *Hum. Comp. Interact.* 22 325–353.

[B13] Bänziger T. Grandjean D. Scherer K. R. (2009). Emotion recognition from expressions in face, voice, and body: the Multimodal Emotion Recognition Test (MERT). *Emotion* 9 691–704. 10.1037/a0017088 19803591

[B14] Bänziger T. Mortillaro M. Scherer K. R. (2012). Introducing the Geneva multimodal expression corpus for experimental research on emotion perception. *Emotion* 12 1161–1179. 10.1037/a0025827 22081890

[B15] Bickmore T. W. Fernando R. Ring L. Schulman D. (2010). Empathic touch by relational agents. *Affect. Comput. IEEE Trans.* 1 60–71. 10.1109/T-AFFC.2010.4

[B16] Bradley M. (1994). Measuring emotion: the self-assessment manikin and the semantic differential. *J. Behav. Ther. Exp. Psychiatry* 25 49–59. 10.1016/0005-7916(94)90063-9 7962581

[B17] Bruckert L. Bestelmeyer P. Latinus M. Rouger J. Charest I. Rousselet G. A. (2010). Vocal attractiveness increases by averaging. *Curr. Biol.* 20 116–120. 10.1016/j.cub.2009.11.034 20129047

[B18] Callaway C. Sima’an K. (2006). Wired for speech: how voice activates and advances the human-computer relationship. *Comput. Linguist.* 32 451–452. 10.1162/coli.2006.32.3.451

[B19] Campanella S. Belin P. (2007). Integrating face and voice in person perception. *Trends Cogn. Sci.* 11 535–543. 10.1016/j.tics.2007.10.001 17997124

[B20] Cao J. Wang H. Hu P. Miao J. (2008). “PAD model based facial expression analysis,” in *Advances in Visual Computing SE*, ed. Bebis G. (Berlin: Springer), 450–459.

[B21] Carroll J. M. Russell J. A. (1996). Do facial expressions signal specific emotions? Judging emotion from the face in context. *J. Pers. Soc. Psychol.* 70 205–218. 10.1037/0022-3514.70.2.205 8636880

[B22] Cha J. Eid M. Rahal L. Saddik A. E. (2008). “HugMe: an interpersonal haptic communication system,” in *Proceedings of the IEEE International Workshop on Haptic Audio visual Environments and Games, 2008*, Ottawa, 99–102. 10.1109/HAVE.2008.4685306

[B23] Courbalay A. Deroche T. Descarreaux M. Prigent E. O’Shaughnessy J. Amorim M. A. (2016). Facial expression overrides lumbopelvic kinematics for clinical judgements about low back pain intensity. *Pain Res. Manag.* 2016:7134825. 10.1155/2016/7134825 27445624 PMC4904626

[B24] Courgeon M. Clavel C. (2013). MARC: a framework that features emotion models for facial animation during human–computer interaction. *J. Multimodal User Interfaces* 7 311–319. 10.1007/s12193-013-0124-1

[B25] de Gelder B. Vroomen J. (2000). The perception of emotions by ear and by eye. *Cogn. Emot.* 14 289–311. 10.1080/026999300378824

[B26] Dolan R. J. Morris J. S. de Gelder B. (2001). Crossmodal binding of fear in voice and face. *Proc. Natl. Acad. Sci. U.S.A.* 98 10006–10010. 10.1073/pnas.171288598 11493699 PMC55568

[B27] Edwards J. Jackson H. J. Pattison P. E. (2002). Emotion recognition via facial expression and affective prosody in schizophrenia: a methodological review. *Clin. Psychol. Rev.* 22 789–832. 10.1016/S0272-7358(02)00130-7 12214327

[B28] Ekman P. Friesen W. V. (1975). *Unmasking the Face: a Guide to Recognizing Emotions From Facial Clues, No. 1968.* Upper Saddle River, NJ: Prentice-Hall.

[B29] Ethofer T. Anders S. Erb M. Droll C. Royen L. Saur R. (2006). Impact of voice on emotional judgment of faces: an event-related fMRI study. *Hum. Brain Mapp.* 27 707–714. 10.1002/hbm.20212 16411179 PMC6871326

[B30] Gaffary Y. Eyharabide V. Martin J.-C. Ammi M. (2013). Clustering approach to characterize haptic expressions of emotions. *ACM Trans. Appl. Percept.* 10 1–20. 10.1145/2536764.2536768

[B31] Gaffary Y. Eyharabide V. Martin J.-C. Ammi M. (2014). The impact of combining kinesthetic and facial expression displays on emotion recognition by users. *Int. J. Hum. Comput. Interact.* 30 904–920. 10.1080/10447318.2014.941276

[B32] Gallace A. Spence C. (2010). The science of interpersonal touch: an overview. *Neurosci. Biobehav. Rev.* 34 246–259. 10.1016/j.neubiorev.2008.10.004 18992276

[B33] Gordon I. Voos A. C. Bennett R. H. Bolling D. Z. Pelphrey K. A. Kaiser M. D. (2013). Brain mechanisms for processing affective touch. *Hum. Brain Mapp.* 34 914–922. 10.1007/s00429-010-0262-0 22125232 PMC6869848

[B34] Haans A. IJsselsteijn W. (2005). Mediated social touch: a review of current research and future directions. *Virtual Real.* 9 149–159. 10.1007/s10055-005-0014-2

[B35] Hertenstein M. J. (2002). Touch: its communicative functions in infancy. *Hum. Dev.* 45 70–94. 10.1159/000048154

[B36] Hofmans J. Mullet E. (2013). Towards unveiling individual differences in different stages of information processing: a clustering-based approach. *Qual. Quant.* 47 455–464. 10.1007/s11135-011-9529-7

[B37] Huisman G. Darriba Frederiks A. Van Dijk B. Hevlen D. Krose B. (2013). “The TaSSt: tactile sleeve for social touch,” in *Proceedings of the 2013 World Haptics Conference*, Tokyo, 211–216. 10.1109/WHC.2013.6548410

[B38] Juslin N. Laukka P. (2003). Communication of emotions in vocal expression and music performance: different channels, same code? *Psychol. Bull.* 129 770–814. 12956543 10.1037/0033-2909.129.5.770

[B39] Kawahara H. Irino T. (2005). “Underlying principles of a high-quality speech manipulation system STRAIGHT and its application to speech segregation,” in *Speech Separation by Humans and Machines SE - 11*, ed. Divenyi P. (Berlin: Springer), 167–180.

[B40] Kawahara H. Morise M. (2011). Technical foundations of TANDEM-STRAIGHT, a speech analysis, modification and synthesis framework. *Sadhana Acad. Proc. Eng. Sci.* 36 713–727.

[B41] Kreiman J. Gerratt B. R. Precoda K. Berke G. S. (1992). Individual differences in voice quality perception. *J. Speech Lang. Hear. Res.* 35 512–520. 10.1044/jshr.3503.512 1608242

[B42] Lecoutre B. Poitevineau J. (2014). *The Significance Test Controversy Revisited: the Fiducial Bayesian Alternative.* Berlin: Springer.

[B43] Lewis M. Haviland-Jones J. M. Barrett L. F. (2010). *Handbook of Emotions.* New York, NY: Guilford Press.

[B44] Massaro W. Egan P. B. (1996). Perceiving affect from the voice and the face. *Psychon. Bull. Rev.* 3 215–221. 10.3758/BF03212421 24213870

[B45] McCabe C. Rolls E. T. Bilderbeck A. McGlone F. (2008). Cognitive influences on the affective representation of touch and the sight of touch in the human brain. *Soc. Cogn. Affect. Neurosci.* 3 97–108. 10.1093/scan/nsn005 19015100 PMC2555465

[B46] McGlone F. Vallbo A. B. Olausson H. Loken L. Wessberg J. (2007). Discriminative touch and emotional touch. *Can. J. Exp. Psychol.* 61 173–183. 10.1037/cjep2007019 17974312

[B47] Mitsunaga N. Miyashita T. Ishiguro H. Kogure K. Hagita N. (2006). “Robovie-IV: a communication robot interacting with people daily in an office,” in *Proceedings of the IEEE/RSJ International Conference on Intelligent Robots and Systems*, Madrid.

[B48] Obrist M. Subramanian S. Gatti E. Long B. Carter T. (2015). “Motions mediated through mid-air haptics,” in *Proceedings of the 33rd Annual ACM Conference on Human Factors in Computing Systems*, Pittsburgh, PA, 2053–2062.

[B49] Oker A. Prigent E. Courgeon M. Eyharabide V. Urbach M. Bazin N. (2015). How and why affective and reactive virtual agents will bring new insights on social cognitive disorders in schizophrenia? An illustration with a virtual card game paradigm. *Front. Hum. Neurosci.* 9:113. 10.3389/fnhum.2015.00133 25870549 PMC4378306

[B50] Olausson H. Wessberg J. Morrison I. McGlone F. Vallbo A. (2010). The neurophysiology of unmyelinated tactile afferents. *Neurosci. Biobehav. Rev.* 34 185–191. 10.1016/j.neubiorev.2008.09.011 18952123

[B51] Oliveira A. Fonseca I. Teixeira M. Simões F. (2005). “A functional measurement approach to the Self-Assessment Manikin,” in *Proceedings of the 21th Annual Meeting of the International Society for Psychophysics in Fechner Day 2005*, Traverse City, MI, 251–256.

[B52] Oliveira A. M. De Sá Teixeira N. Oliveira M. P. Breda S. J. Da Fonseca I. (2007). Algebraic integration models of facial features of expression: a case made for pain. *Theor. Model.* 12 167–180.

[B53] Park Y. (2010). “CheekTouch: an affective interaction technique while speaking on the mobile phone,” in *Proceedings of the Human Factors in Computer Systems*, Atlanta, GA, 3241–3246.

[B54] Patel T. (2013). *The Handbook of Touch.* Berlin: Springer Publishing.

[B55] Pereira T. Oliveira A. Fonseca I. B. (2016). Brain activation follows adding-type integration laws: brain and rating responses in an integration task with pairs of emotional faces. *Univ. Psychol.* 15 25–62. 10.11144/Javeriana.upsy15-3.bafa

[B56] Pierre-Yves O. (2003). The production and recognition of emotions in speech: features and algorithms. *Int. J. Hum. Comput. Stud.* 59 157–183. 10.1016/S1071-5819(02)00141-6

[B57] Prigent E. Amorim M.-A. Leconte P. Pradon D. (2014). Perceptual weighting of pain behaviours of others, not information integration, varies with expertise. *Eur. J. Pain* 18 110–119. 10.1002/j.1532-2149.2013.00354.x 23821536

[B58] Russell J. Mehrebian A. (1977). Evidence for a three-factor theory of emotions. *J. Res. Pers.* 11 273–294. 10.1016/0092-6566(77)90037-X

[B59] Saldien J. Goris K. Yilmazyildiz S. Verhelst W. Lefeber D. (2008). On the design of the huggable robot probo. *J. Phys. Agents* 2 3–11.

[B60] Scherer K. (2003). Vocal communication of emotion: a review of research paradigms. *Speech Commun.* 40 227–256. 10.1016/S0167-6393(02)00084-5

[B61] Scherer K. R. Bänziger T. Roesch E. (2010). *A Blueprint for Affective Computing: a Sourcebook and Manual.* Oxford: Oxford University Press.

[B62] Shackman E. Pollak S. D. (2005). Experiential influences on multimodal perception of emotion. *Child Dev.* 76 1116–1126. 10.1111/j.1467-8624.2005.00901.x 16150006

[B63] Silva D. Oliveira A. M. (2016). Do faces and body postures integrate similarly for distinct emotions, kinds of emotion and judgment dimensions? *Univ. Psychol.* 15 1–21.

[B64] Tsalamlal M. Ouarti N. Martin J.-C. Ammi M. (2015). Haptic communication of dimensions of emotions using air jet based tactile stimulation. *J. Multimodal User Interfaces* 9 69–77. 10.1007/s12193-014-0162-3

[B65] Tsalamlal M. Y. Ouarti N. Ammi M. (2013). “Psychophysical study of air jet based tactile stimulation’,” in *Proceedings of the IEEE World Haptics Conference (WHC)*, Piscataway, NJ, 639–644.

[B66] Tsetserukou D. Neviarouskaya A. (2010). “World’s first wearable humanoid robot that augments our emotions,” in *Proceedings of the 1st Augmented Human International Conference - AH’10 2010*, New York, NY, 1–10.

[B67] Vanderborght B. Simut R. Saldien J. Pop C. Rusu A. S. Pintea S. (2012). Using the social robot probo as a social story telling agent for children with ASD. *Interact. Stud.* 13 348–372. 10.1075/is.13.3.02van

[B68] Vidotto G. Massidda D. Noventa S. (2010). Averaging models: parameters estimation with the r-average procedure. *Psicol. Int. J. Methodol. Exp. Psychol.* 31 461–475.

[B69] Yohanan S. MacLean K. E. (2011). The role of affective touch in human-robot interaction: human intent and expectations in touching the haptic creature. *Int. J. Soc. Robot.* 4 163–180. 10.1007/s12369-011-0126-7

[B70] Zalinski J. Anderson N. H. (1989). “Measurement of importance in multiattribute models,” in *Conditioning, Cognition, and Methodology: Contemporary Issues in Experimental Psychology*, ed. Sidowski J. B. (Lanham, MD: University Press of America), 177–215.

